# Memantine protects the cultured rat hippocampal neurons treated by NMDA and amyloid β1–42

**DOI:** 10.3389/fnins.2023.1269664

**Published:** 2023-12-08

**Authors:** Nataliia M. Rozumna, Vita V. Hanzha, Elena A. Lukyanetz

**Affiliations:** Department of Biophysics of Ion Channels, Bogomoletz Institute of Physiology, National Academy of Sciences of Ukraine, Kyiv, Ukraine

**Keywords:** Alzheimer’s disease, beta amyloid, cell culture techniques, hippocampus, neurons, neuroprotection, NMDA, memantine

## Abstract

Alzheimer’s disease (AD) is a devastating neurodegenerative condition with no effective treatments. Recent research highlights the role of NMDA receptors in AD development, as excessive activation of these receptors triggers excitotoxicity. Memantine, an NMDA receptor antagonist, shows promise in curbing excitotoxicity. What sets our study apart is our novel exploration of memantine’s potential to protect hippocampal neurons from neurotoxicity induced by NMDA and amyloid β1–42, a hallmark of AD. To achieve this, we conducted a series of experiments using rat hippocampal cell cultures. We employed Hoechst and propidium iodide double staining to assess neuronal viability. Analyzing the viability of neurons in normal conditions compared to their status after 24 h of exposure to the respective agents revealed compelling results. The incubation of hippocampal neurons with NMDA or amyloid β1–42 led to a more than twofold increase in the number of apoptotic and necrotic neurons. However, when memantine was co-administered with NMDA or amyloid β1–42, we witnessed a notable augmentation in the number of viable cells. This unique approach not only suggests that memantine may act as a neuroprotective agent but also emphasizes the relevance of hippocampal neuron cultures as valuable models for investigating excitotoxicity and potential AD treatments.

## Graphical abstract

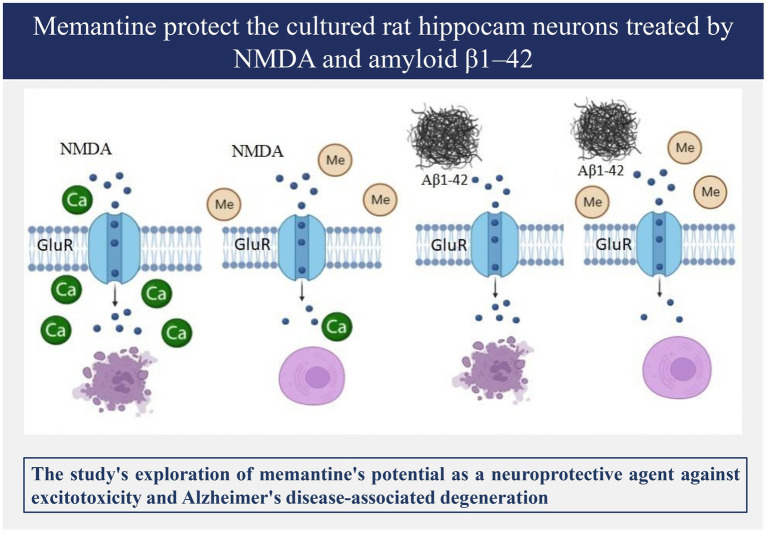

## 1 Introduction

Alzheimer’s disease (AD) stands as a multifaceted neurodegenerative disorder, predominantly distinguished by the accumulation of senile plaques and neurofibrillary tangles within the cerebral tissue. These hallmark features primarily consist of insoluble beta-amyloid (Aβ) deposits and hyperphosphorylated tau protein (Bloom, 2014; Breijyeh and Karaman, 2020; Busche and Hyman, 2020; Tyshchenko and Lukyanetz, 2020; Ashrafian et al., 2021). The formation of these plaques is intricately linked to the proteolytic processing of the amyloid precursor protein (APP) by β- and γ-secretases. In contrast, neurofibrillary tangles manifest as intraneuronal aggregates rich in hyperphosphorylated tau protein. Recent research endeavors have illuminated the diverse array of Aβ forms and their unique impacts on cellular structures (Kravenska et al., 2020). Notably, soluble Aβ oligomers have surfaced as highly neurotoxic entities, significantly disrupting synaptic function and precipitating cognitive impairments (Wang and Reddy, 2017; Battaglia et al., 2023; Polyák et al., 2023). Aβ also exerts significant influence on neuronal function, affecting impulse activity and other crucial cellular processes (Yavorsky et al., 2023). Intermediate forms known as protofibrils, bridging oligomers and fibrils, have been identified as potent instigators of inflammation and disruptors of intracellular signaling. Insoluble fibrillar Aβ, a hallmark of AD seen in amyloid plaques, inflicts physical harm upon cells, incites inflammation, and activates immune responses. Nevertheless, contemporary studies have underscored that it is the soluble Aβ oligomers, rather than the insoluble plaques, which primarily drive neurodegeneration and synaptic dysfunction in AD (Citron, 2010). These oligomers play a pivotal role in inducing clinically significant cognitive impairments, even before conspicuous neuronal loss emerges (Tanaka et al., 2022b; Liu et al., 2023; Tanaka et al., 2023). The hippocampus, a region vital for learning and memory, becomes particularly susceptible to AD pathology, with up to 80% of its neurons at risk of demise during the course of the disease, thereby substantially contributing to cognitive deterioration. Recent insights further hint at the interaction between soluble Aβ oligomers and glutamatergic receptors, especially the N-methyl-D-aspartate (NMDA) receptor, in the pathogenesis of AD (Findley et al., 2019; Penke et al., 2020; Xu et al., 2023). Glutamate, the principal excitatory neurotransmitter in the brain, is widely distributed in the neocortex and hippocampus. In normal physiological conditions, glutamate release into the synaptic cleft activates NMDA receptors, thereby regulating neuronal activity through calcium ion influx (Gonzalez et al., 2015; Carvajal et al., 2016). However, in the presence of excess glutamate or Aβ-induced excitotoxicity, these NMDA receptors are prone to overactivation, thereby setting off a chain of degenerative processes in neurons, encompassing oxidative stress, calcium overload, and apoptosis (Mody and MacDonald, 1995; Correa et al., 2022). In stroke, activation of NMDARs can lead to excitotoxicity and neuronal damage (Wu and Tymianski, 2018). Altered NMDAR function has been associated with epileptic seizures. Both hypoactivity and hyperactivity of NMDARs can contribute to the dysregulation of synaptic transmission, leading to the development and progression of epilepsy (Ghasemi and Schachter, 2011). Dysfunction of NMDARs, particularly the subunit NR2A, has been implicated in the pathophysiology of schizophrenia (Nakazawa and Sapkota, 2020). Impaired NMDAR-mediated synaptic plasticity and hypofunction of NMDARs contribute to the cognitive deficits observed in this disorder (Finke et al., 2012).

Taking into attention the pivotal role of hippocampal neurons in AD pathogenesis and the intricate involvement of glutamatergic signaling, there arises a critical need to explore potential neuroprotective strategies targeting NMDA receptors. Among these strategies, memantine, serving as an NMDA receptor antagonist, has displayed notable promise in both preclinical investigations and clinical trials, accentuating its potential therapeutic significance not only in AD but also in other neurological disorders (Tanaka et al., 2020; Kim et al., 2021; Delussi et al., 2022; Tanaka et al., 2022a).

Given this backdrop, our hypothesis posits that the interaction between soluble Aβ oligomers and NMDA receptors, along with other glutamate-related proteins, plays a pivotal role in the pathogenesis of AD. Our study seeks to assess and compare the impact of two neurodegenerative agents, NMDA and Aβ, on the viability of hippocampal neurons within a cell culture model of AD. Additionally, we aim to investigate the potential neuroprotective properties of memantine (3,5-dimethyladamantan-1-amine), an NMDA receptor antagonist, on neurons exposed to these detrimental agents. Our investigation seeks to illuminate the potential involvement of glutamate receptors in the pathogenesis of AD, offering a fresh perspective on the interplay between these neurotoxic agents and neurodegeneration.

## 2 Materials and methods

The following section outlines the methodologies and procedures employed in our study to investigate the neuroprotective potential of memantine in the context of Alzheimer’s disease (AD). Through a series of carefully designed experiments, we sought to examine the impact of two neurodegenerative agents, NMDA and Aβ1–42, on hippocampal neuron viability within a cell culture model. Additionally, we aimed to assess the potential therapeutic efficacy of memantine, an NMDA receptor antagonist, in mitigating the neurotoxic effects of these agents.

All experimental procedures followed the European Commission Directive (86/609/EEC) and ethical guidelines of the International Association for the Study of Pain and were approved by the local Animal Ethics Committee of the Bogomoletz Institute of Physiology (Kyiv, Ukraine). All efforts were made to minimize the number and suffering of animals used.

We used the method described in detail previously (Korol et al., 2008; Kravenska et al., 2012, 2016) to prepare hippocampal neurons’ primary dissociated culture. We took neurons for the experiment on day 12–13 of cultivation (see Supplementary Figure S1). It was during this period of neuronal development in our cultures hippocampal neurons demonstrated the presence of well-developed dendritic spines and the formation of synaptic connections. This was confirmed using techniques such as electrophysiology and fluorescent Ca^2+^ measurements in our laboratory (Rozumna et al., 2020; Shkryl, 2022; Pendeliuk and Melnick, 2023). And as we know from the literature (Harrill et al., 2015; Napoli and Obeid, 2016; Karapetyan et al., 2022) and protocols for working with cultures (Vicario-Abejón, 2004; Yang et al., 2017; Sahu et al., 2019) the neuronal properties, such as the formation of functional synapses, the ability to generate action potentials, and the development of dendritic arborization, serve as indicators of neuronal maturation.

Hippocampal cultures are widely used to study the mechanisms and strategies for treating the central nervous system’s disorders, including AD (Calvo-Rodriguez et al., 2019; Coelho et al., 2019; Hanzha et al., 2023). In our studies, the AD model was obtained by 24-h incubation of hippocampal culture neurons with 2 μM Aβ1–42 (Sigma-Aldrich, St. Louis, MO, United States). To study the effect of non-competitive low-affinity NMDA receptor antagonist - memantine (Sigma-Aldrich, United States) and the NMDA (Sigma-Aldrich, United States), an agonist of the receptors on the viability of neurons, we incubated hippocampal cell culture with these compounds separately and together with Aβ1–42 reagents (see Supplementary Figure S1). The concentrated Aβ1–42 solution was prepared on dimethyl sulfoxide (DMSO) and stored at −20°C. The final concentration of DMSO did not exceed 0.5% in media.

In our study, we utilized different groups to investigate the distinct effects and interactions of various substances or combinations. These group classifications were based on the specific objectives of our research and the substances involved:

**Group 1:**

- Control: this group served as the baseline or reference group to provide a comparison point for the other groups.
- Memantine: this group was exposed to memantine to assess its individual impact.
- NMDA: NMDA was administered to understand its effects on its own.
- NMDA + Memantine: this group was established to examine the potential interplay between memantine and NMDA, assessing whether memantine could provide neuroprotection against excitotoxicity induced by NMDA.

**Group 2:**

- Control: similar to Group 1, this control group acted as a reference for the specific substances involved in Group 2.
- Memantine: this group received memantine to assess its effects independently.
- Aβ: the introduction of Aβ1–42 aimed to study its individual effects.
- Aβ + Memantine: this group was included to evaluate any potential protective effect of memantine against the harmful actions of Aβ.

The inclusion of these different groups allowed us to systematically examine the impact of individual substances and potential synergistic effects when they were used together. This approach was crucial for addressing our research questions and achieving a comprehensive understanding of the interactions and outcomes associated with these substances. Experiments were repeated at least four different times.

The selection of treatment dosages for NMDA, Aβ1–42 and memantine in our scientific work was a carefully considered process, grounded in a synthesis of our previous experiments, existing literature, consensus protocols, and the specific objectives of our research. To determine an effective toxic dose of Aβ1–42, we previously conducted a series of experiments in which we evaluated the effects of Aβ β1–42 at doses of 1, 2, 5, and 10 μM. Our analysis indicated that Aβ1–42 had a low toxic effect at a dose of 1 μM, moderately decreased cell viability at doses of 2 μM, and significantly decreased cell viability at doses of 5 and 10 μM. Based on these results and a comprehensive review of the existing literature (Danysz and Parsons, 2012; Calvo-Rodriguez et al., 2016; Kodis et al., 2018; Caballero et al., 2022), we decided to use Aβ1–42 at a dose of 2 μM for the remaining experiments. The dosage of NMDA was determined through a review of pertinent scientific literature and research articles. Various studies have employed a range of NMDA concentrations, typically falling between 10 μM and 500 μM, depending on their specific objectives and models (Calvo et al., 2015; Calvo-Rodríguez et al., 2017; Caballero et al., 2022). By systematically reviewing and analyzing these articles, we gathered valuable insights into dosing strategies and the efficacy outcomes associated with this drug. The dosage we selected for our research (10 μM), in accordance with the literature, falls within the range known to produce the desired effects without inducing excessive toxicity. The dosage selection for memantine in our study was based on existing literature, preliminary experiments, and the specific goals of our research. Several cell culture-based studies have shown that memantine exhibits pharmacological activity at doses ranging from 1 μM to 100 μM, depending on the specific cell types (Alley et al., 2010; Danysz and Parsons, 2012; Hirano et al., 2019; Kolcheva et al., 2021; Boehringer et al., 2023; de Wet et al., 2023). Consequently, we chose a single dose of 50 μM for memantine in our current study.

The number of living, apoptotic, and necrotic cells was determined by double staining with Hoechst 33258 (Sigma-Aldrich, United States) and propidium iodide (PI, Sigma-Aldrich, United States) (see Supplementary Figure S1).

Images (1,024 × 1,024 pixels) were acquired using an FV1000-BX61WI laser scanning microscope (Olympus, Japan) using a 20 × objective (see Supplementary Figure S1). The excitation laser wavelengths were 352/405 nm (using Hoechst 33258) and 543 nm (using PI). For five spatially distant areas of each specimen, contained from 100 to 300 cells, three images were obtained: the phase contrast signal (to distinguish neurons from glial cells), Hoechst (to detect living and apoptotic cells), and a PI (to detect cell necrosis). Cells were counted using the ImageJ cell counter plugin for brain sections (https://imagej.nih.gov/ij/plugins/cell-counter.html, National Institutes of Health, Bethesda, MD, United States) and classified as follows:

- living cells (normal nuclei: blue chromatin with organized structure);
- apoptotic cells (condensed or fragmented nuclei with bright blue chromatin);
- necrotic cells (red, enlarged nuclei with smooth normal structure).

Based on the staining we count the total number of cells in each category, which includes living, apoptotic, and necrotic cells. Then the relative number of cells (living, apoptotic, and necrotic) was calculated by dividing the number of cells in each category by the total number of cells.

Example calculation:

- % Living Cells = (Number of Living Cells/Total Number of Cells) × 100.
- % Apoptotic Cells = (Number of Apoptotic Cells/Total Number of Cells) × 100.
- % Necrotic Cells = (Number of Necrotic Cells/Total Number of Cells) × 100.

We performed a one-way analysis of variance ANOVA to compare cell viability between experimental groups using Origin software (OriginLab Corporation, Northampton, MA, United States).

## 3 Results

*In vitro* experiments on hippocampal neurons in culture were devoted to investigating the neuroprotective role of the non-competitive low-affinity NMDA receptor antagonist memantine on these cells’ viability in modeling excitotoxicity and AD. To this end, we conducted six series of experiments: (1) Control conditions; (2) Introduction of memantine; (3) Introduction of NMDA; (4) Simultaneous administration of NMDA and memantine; (5) Introduction of Aβ1–42; (6) Simultaneous administration of memantine and Aβ1–42.

**Figure 1:**
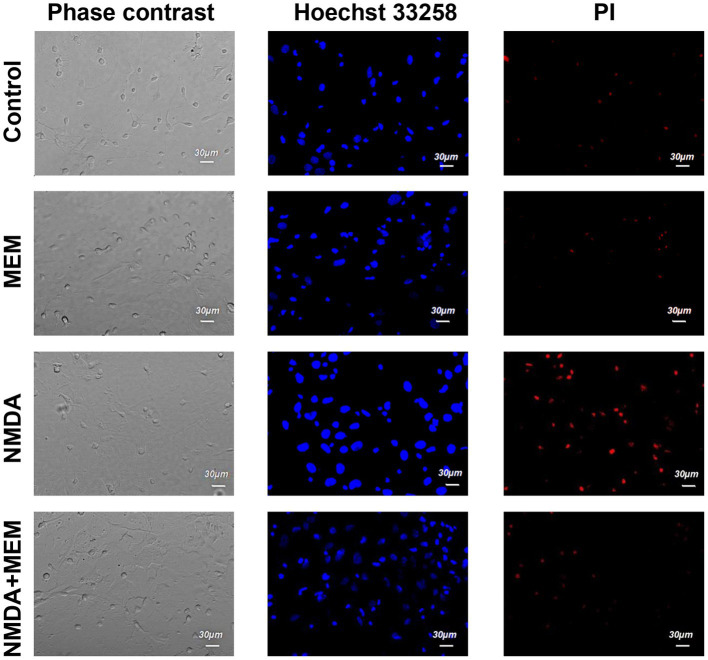
The effect of memantine on the viability status of hippocampal neurons treated by NMDA. The images of hippocampal culture neurons are shown that were incubated in control (control) media, in the presence of an activator of NMDA type glutamate receptors (NMDA), and in the presence of NMDA and memantine (NMDA + MEM). The images were obtained in phase-contrast mode (phase-contrast), using Hoechst 33258 dye to determine living and apoptotic cells (Hoechst 33258) and propidium iodide (PI) dye to determine the necrotic cells.

**Figure 2:**
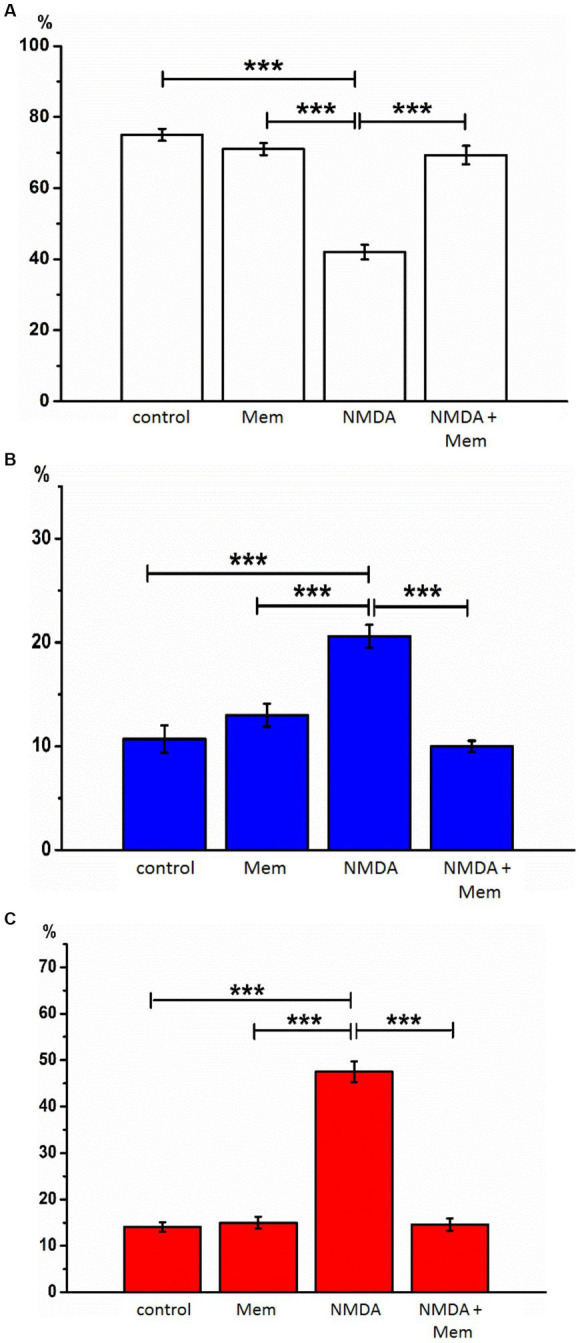
The effect of memantine on the relative numbers of hippocampal neurons treated by NMDA at different state. The relative numbers of living cells **(A)**, cells with signs of apoptosis **(B)**, and necrosis **(C)** in the culture of rat hippocampal neurons with the addition of NMDA or/and memantine. ***p < 0.001.

The cells were then stained twice with two DNA-binding dyes (Hoechst and PI), and we assessed the viability of the neurons by counting them using confocal laser scanning microscopy, comparing the fluorescence of the cells under control conditions and after 24 h of incubation with the above these reagents. In control samples of rat hippocampal cell culture, the vast majority of cells (average 75.1% ± 1.72) did not show any signs of pathological changes, and we evaluated them as alive (Figure 1). The nuclei of such neurons uniformly accumulated the nucleophore Hoechst 33258 and were 5 characterized by faint blue fluorescence. They had clear contours and an intact nuclear architecture. The nuclei of a relatively small proportion of neurons in control samples (10.7% ± 1.34) with bright blue fluorescence showed physiological changes in nuclear chromatin, such as its condensation and fragmentation, which are all signs of apoptosis. Those neurons, which under control conditions, were stained with PI and gave red fluorescence indicating their necrotic degeneration, amounted to 14.1% ± 0.98 on average in the group (Figures 1, 2).

At first, we conducted a series of experiments to test whether memantine would prevent apoptosis and necrosis of neurons caused by excessive activation of NMDA receptors in our experimental conditions. To do this, we treated cultures with memantine (50 μM) and simultaneously with NMDA (10 μM) for 24 h.

After incubation with NMDA receptor antagonist memantine (50 μM, 24 h) in the second series of experiments, we found that the proportion of living neurons averaged 71.1% ± 1.70, apoptotic - 13.0% ± 1.10, necrotized - 15.0% ± 1.30 of the total number of studied neurons. We did not detect statistically significant changes in this group of cells compared with the control, so memantine itself had no side effects on the hippocampal culture cells (Figure 2).

After incubation of hippocampal cell culture samples in NMDA containing medium (10 μM, 24 h), the number of living cells was approximately one-third of the control group (mean 31.9% ± 2.18). Neurons with apoptotic changes in these conditions were observed in 20.6% ± 1.11 of cases. The group of cells with signs of necrosis was more numerous than in control samples by approximately 33% (*p* < 0.001), on average, their number was 47.5% ± 2.21 (Figure 2). Thus, the introduction of NMDA into the culture medium induced intense death of hippocampal neurons. In half of the cells of the studied samples, there were pronounced pathological changes.

After co-incubation of hippocampal neurons with NMDA (10 μM, 24 h) and memantine (50 μM, 24 h), the proportion of living neurons relative to the total number of cells was 75.4%, ± 1.72 apoptotic - 10% ± 0.54, necrotic - 14.6% ± 1.31 (Figure 2). Comparing these results with those obtained during incubation with the addition of alone NMDA, the number of living cells increased by 43.5% (*p* < 0.001), the number of apoptotic and necrotic decreased by 10% (*p* < 0.001) and 32% (*p* < 0.001), respectively. The latter indicates that memantine in our experimental conditions had a protective effect against excitotoxicity caused by NMDA’s addition in the culture medium.

In the following series of experiments, we investigated memantine’s neuroprotective ability against the neurotoxic effects of Aβ1–42. To do this, we treated cultures with Aβ1–42 (2 μM) and simultaneously with memantine (50 μM), the specific NMDA receptor antagonist, for 24 h.

**Figure 3:**
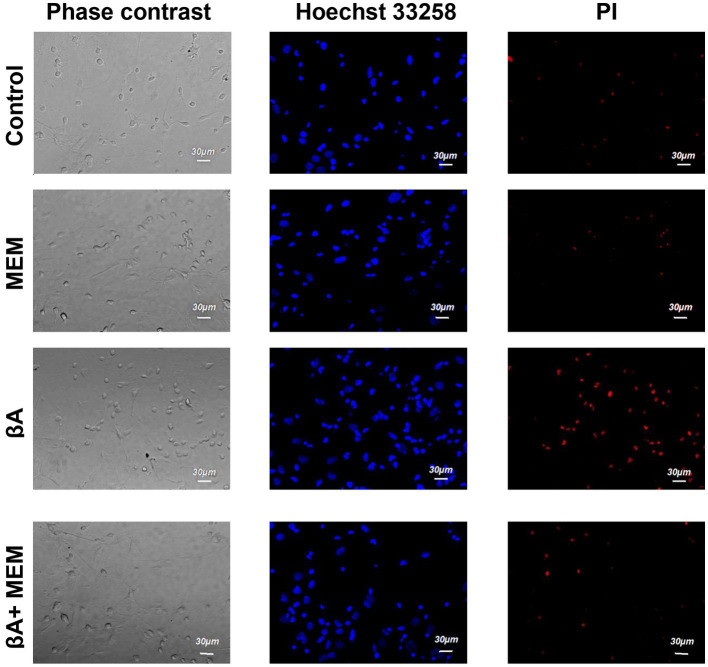
The effect of memantine on the viability status of hippocampal neurons treated by Aβ1–42. The images of hippocampal culture neurons are shown that were incubated in the presence of memantine (MEM), in the presence of Aβ1–42 (Aβ), and in the presence of Aβ1–42 and memantine (Aβ + MEM). The images were obtained as described in Figure 1.

**Figure 4:**
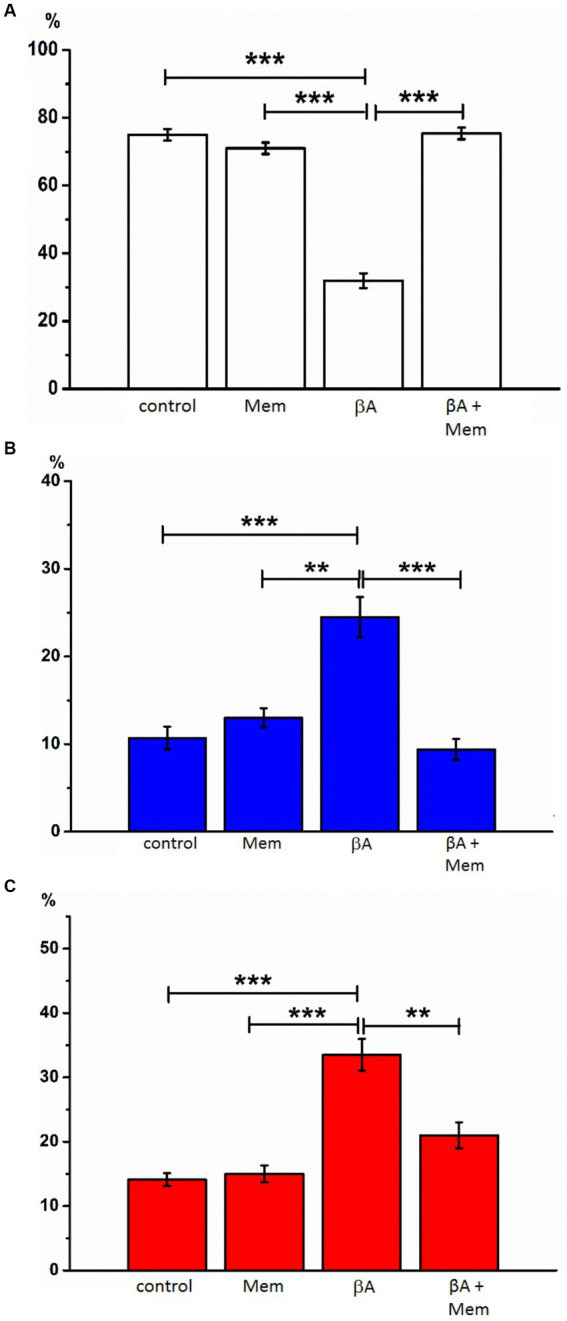
The effect of memantine on the relative numbers of hippocampal neurons treated by Aβ1-42 at different state. The relative numbers of living cells **(A)**, cells with signs of apoptosis **(B)**, and necrosis **(C)** in the culture of rat hippocampal neurons with the addition of Aβ1–42 and memantine. **p < 0.01, ***p < 0.001.

Introduction of Aβ1–42 (2 μM, 24 h, AD simulation) into the incubation medium resulted in a decrease in normal cytological cells (average 42.0% ± 2.08). Neurons with signs of apoptosis in these conditions were observed in approximately 24.5% ± 2.29. The group of neurons with necrotic changes was also more numerous than in control samples (on average, their number was 33.5% ± 2.45) (Figures 3, 4). Thus, Aβ1–42 added to the culture medium caused a neurotoxic effect and induced hippocampal neurons’ death.

With the simultaneous introduction of Aβ1–42 and memantine in the incubation media, the results indicated preserving a larger number of living cells than under conditions of alone action of Aβ1–42. The relative number of neurons without signs of apoptosis or necrosis, in this case, was much more than half of the analyzed cells (average 69.3% ± 2.60) and statistically significant differed from the corresponding values in the alone action of Aβ1–42 (Figure 4). The number of neurons with apoptotic changes in the described group was approximately the same as in the control conditions (9.4% ± 1.20 of the total number), and this indicator was approximately twice less than the same value in the above group of cells with alone action of Aβ1–42. The addition of memantine provided a decrease in the number of apoptotic cells (*p* < 0.001). The relative number of cells with clear signs of necrosis in these two groups also differed by approximately by 1.5 times (when memantine was added simultaneously with Aβ1–42 in the media, this indicator averaged was 21.0% ± 2.00, *p* < 0.01). That is, the addition of memantine suspended the pathological effect of Aβ1–42 on hippocampal culture neurons.

The observed changes in cell viability in our study carry significant biological implications that shed light on the pathophysiology of Alzheimer’s disease (AD) and potential mechanisms underlying the neuroprotective effects of memantine. Our findings revealed a striking contrast in cell viability. Exposure to NMDA or Aβ1–42 led to a substantial increase in apoptotic and necrotic neurons, underscoring the neurotoxic nature of these agents. This aligns with the prevailing understanding of AD, where the accumulation of Aβ and NMDA receptor overactivation plays a crucial role in the loss of neuronal integrity. What sets our study apart is the remarkable increase in viable cells when memantine, an NMDA receptor antagonist, was co-administered with NMDA or Aβ1–42. This suggests that memantine holds potential as a neuroprotective agent against the detrimental effects of these neurotoxic agents. By mitigating the excitotoxicity driven by NMDA receptors, memantine may help protect hippocampal neurons from the damage associated with AD.

Moreover, our use of hippocampal neuron cultures in this context emphasizes the significance of this model in exploring excitotoxicity and potential therapeutic interventions for AD. The outcomes of our study not only contribute to the understanding of the pathophysiology of AD but also offer insights into the mechanisms by which memantine exerts its neuroprotective effects. In a broader context, this research advances our knowledge of the interplay between NMDA receptors, Aβ toxicity, and potential therapeutic interventions. While AD remains a complex challenge, our study provides a promising avenue for further investigations into the development of treatments that may ultimately alleviate the burden of this devastating condition.

## 4 Discussion

Research in AD and the development of new substances like memantine are critically important. AD is a leading cause of dementia, with a growing global impact. Understanding the disease’s mechanisms is essential for developing effective treatments. Memantine shows promise in mitigating cognitive decline. These studies have the potential to enhance our understanding of AD’s complex mechanisms and offer innovative therapeutic approaches.

Our study delves into the intricate realm of AD and explores the potential neuroprotective properties of memantine, specifically in the context of NMDA receptor activation and Aβ1–42 exposure. The primary aim was to evaluate the impact of memantine on neuronal viability in the presence of these neurotoxic agents.

A growing body of evidence suggests that some amyloid-induced neuron degeneration is associated with the overactivation of NMDA (Wang and Reddy, 2017; Companys-Alemany et al., 2022). Several publications highlight memantine as a potential therapeutic agent for AD (Bond et al., 2012; Olivares et al., 2012; Tan et al., 2014; Marotta et al., 2020; Majidazar et al., 2022; Saeedi and Mehranfar, 2022). Based on this, we first start to test whether memantine would prevent apoptosis and necrosis of neurons caused by excessive activation of NMDA receptors in our experimental conditions. To do this, we conducted a series of experiments with the addition of NMDA, the actual agonist of these receptors, and the simultaneous introduction of memantine for 24 h into the incubation medium. The results of our studies showed that under the influence of NMDA, the number of living cells significantly decreased, and at the same time, there was a significant death of neurons by apoptosis and necrosis. Thus, in our experiments we reproduced the excitotoxicity associated with the overactivation of glutamatergic receptors. The latter is a cascade of degenerative reactions in neurons related to a massive influx of calcium ions into neurons. Calcium ions activate proteases and phospholipase A2, disrupting oxidative phosphorylation, inducing mitochondrial dysfunction, and releasing arachidonic acid, which contributes to neuronal damage and death through apoptosis or necrosis (Danysz et al., 2000; Rozumna et al., 2020).

Among the drugs targeting the glutamatergic system, memantine, an NMDA receptor antagonist, stands out. Memantine rapidly binds and reversibly interacts with NMDA receptors, distinguishing it favorably from other NMDA receptor antagonists and contributing to its efficacy. Acting as a non-competitive, low-affinity, voltage-dependent antagonist, memantine blocks the cation channel during resting state, thereby interrupting the cascade of neurodegeneration caused by calcium influx and oxidative stress in postsynaptic neurons (Majláth et al., 2016; Majidazar et al., 2022; Ippoliti et al., 2023). This mechanism helps to stop this type of receptors’ excessive activity while maintaining the possibility of conducting physiological glutamatergic receptor-effector reactions (Danysz et al., 2000; Volbracht et al., 2006; Folch et al., 2018). Experiments have consistently demonstrated that treatment with memantine leads to a reduction in both the volume and extent of neuronal damage, suggesting its potential neuroprotective properties. The effectiveness of memantine has been observed in various experimental models, including cerebral infarction (Görgülü et al., 2000), hemorrhage, chronic ischemia, traumatic injury (Padovani et al., 2023; Thakral et al., 2023), in cortical culture neurons (Lopes et al., 2013), granular cells of the cerebellum (Volbracht et al., 2006) and retinal ganglion cells (Hassan et al., 2022). Under the conditions of our experimental model after co-incubation of hippocampal neurons with NMDA and memantine the number of living cells increased, and the number of apoptotic and necrotic cells decreased compared to the values obtained with the isolated addition of NMDA alone, approaching to the control values. Нence, memantine had a protective effect against excitotoxicity caused by NMDA addition in the culture medium in our experimental conditions. Thus, our experimental studies’ results as well as the data of other researchers provided evidence supporting the promising therapeutic strategy of inhibiting NMDA receptors in AD. By reducing the activity of this receptors involved in triggering the apoptosis cascade, it is likely to slow down neurodegeneration and alleviate disease symptoms by enhancing the viability of specific neuronal populations.

Based on the fact that, according to the literature, the neurotoxicity of Aβ1–42 can be mediated by the excitotoxicity of glutamate (Yu et al., 2023; Zhang et al., 2023), and that memantine, as an antagonist of glutamate NMDA receptors, by blocking them prevents neuronal death as a result of this excitotoxicity, it is reasonable to hypothesize that the memantine may have a neuroprotective effect against the neurotoxicity of Aβ1–42. To test this hypothesis we conducted our second series of experiments where we investigated memantine’s neuroprotective ability against the neurotoxic effects of Aβ1–42. Incubation our cultures with Aβ1–42 within 24 h resulted in a decrease in the number of normal cytological cells, and there were more neurons with apoptotic and necrotic changes than in the control samples. Thus, Aβ1–42 added to the culture medium induced a neurotoxic effect and led to the death of hippocampal neurons. This confirms that we have reproduced the conditions of amyloid neurotoxicity in our experiments, Other researchers obtained similar data on the toxic effects and reduced viability of cells under the action of Aβ1–42, for example, in the hippocampus’s organotypic culture (Arbo et al., 2017), in the primary culture of the hippocampus (Hooshmandi et al., 2020). In these studies, after incubating these cells with Aβ, the amyloid-induced morphological changes, such as shrinkage of neuronal bodies, increased the number of dead cells, and cell fragments were observed. Simultaneously adding Aβ1–42 and memantine to the incubation medium resulted in the preservation of a greater number of living cells and a reduction in the number of apoptotic and necrotic cells compared to when only Aβ1–42 was present. Taken together, our results demonstrate that blocking NMDA receptors in hippocampal neuron culture model of AD leads to the regulation of potent and prolonged survival pathways, preventing apoptosis and necrosis. There are not many studies similar to ours on cell cultures at present, but they are, to some extent, consistent with our results. Thus, in *in vitro* experiments, memantine has demonstrated the ability to neutralize beta-amyloid effects, including its soluble oligomers, in cultures of cortical and hippocampal neurons (Lacor et al., 2007; Companys-Alemany et al., 2022; Wilcox et al., 2022). Previously it was also found that memantine prevents pathological apoptosis and neuronal loss in the hippocampus when co-administered with Aβ1–40 (Miguel-Hidalgo et al., 2002; Chayrov et al., 2022) i.e., it has a neuroprotective effect. Memantine has also shown procognitive effects in preclinical AD models. In rat experiments, memantine prevented short-term memory deficits caused by Aβ1–40, improved long-term memory consolidation impaired by soluble beta-amyloid, enhanced spatial memory in animals injected with Aβ1–42 oligomers, and improved performance in other behavioral memory tests (Kruchenko et al., 2014; Tyshchenko and Lukyanetz, 2017; Gorbachenko and Lukyanetz, 2020).

The outcomes of our experiments highlight the potential of memantine as a neuroprotective agent against NMDA and Aβ-induced neurotoxicity. This not only reinforces the significance of NMDA receptors but also opens avenues for innovative treatments targeting excitotoxicity. We posit that memantine’s efficacy suggests promising prospects for ameliorating cognitive decline in AD patients. This study paves the way for future investigations into the intricate interactions within the AD pathogenesis. Exploring memantine’s effectiveness in complex conditions remains an avenue brimming with potential.

We acknowledge that our study has certain limitations, including its reliance on *in vitro* cell culture models. However, its merits lie in the clarity of results and the promise it holds for further exploration. The potential translation of our findings into clinical practice is a beacon of hope for AD patients, offering the prospect of interventions that may safeguard cognitive function.

## 5 Conclusion

In summary, memantine has the potential to reduced neurons damage caused by NMDA and Aβ1–42 based on our study. This finding advances our understanding of AD and might carry translational implications for AD treatment. However, further research to explore NMDA and Aβ interactions is needed to understand the impact of memantine on AD model animal and AD patient.

## Data availability statement

The raw data supporting the conclusions of this article will be made available by the authors, without undue reservation.

## Ethics statement

The animal study was approved by Ganna V. Sotkis, Bogomoletz Institute of Physiology, National Academy of Sciences of Ukraine. The study was conducted in accordance with the local legislation and institutional requirements.

## Author contributions

NR: Formal analysis, Investigation, Writing – original draft, Software, Visualization. VH: Formal analysis, Investigation, Software, Writing – original draft. EL: Conceptualization, Data curation, Funding acquisition, Methodology, Project administration, Resources, Supervision, Validation, Writing – review & editing.

## References

[ref1] Alley G. M. Bailey J. A. Chen D. M. Ray B. Puli L. K. Tanila H.. (2010). Memantine lowers amyloid-beta peptide levels in neuronal cultures and in APP/PS1 transgenic mice. J. Neurosci. Res. 88, 143–154. doi: 10.1002/JNR.22172, PMID: 19642202 PMC2783840

[ref2] Arbo B. D. Hoppe J. B. Rodrigues K. Garcia-Segura L. M. Salbego C. G. Ribeiro M. F. (2017). 4′-Chlorodiazepam is neuroprotective against amyloid-beta in organotypic hippocampal cultures. J. Steroid Biochem. Mol. Biol. 171, 281–287. doi: 10.1016/j.jsbmb.2017.04.010 28442392

[ref3] Ashrafian H. Zadeh E. H. Khan R. H. (2021). Review on Alzheimer’s disease: Inhibition of amyloid beta and tau tangle formation. Int. J. Biol. Macromol. 167, 382–394. doi: 10.1016/j.ijbiomac.2020.11.192 33278431

[ref4] Battaglia S. Nazzi C. Thayer J. F. (2023). Fear-induced bradycardia in mental disorders: Foundations, current advances, future perspectives. Neurosci. Biobehav. Rev. 149:105163. doi: 10.1016/J.NEUBIOREV.2023.105163, PMID: 37028578

[ref5] Bloom G. S. (2014). Amyloid-β and Tau. JAMA Neurol. 71:505. doi: 10.1001/jamaneurol.2013.5847 24493463 PMC12908160

[ref6] Boehringer S. C. Johnston T. V. Kwapisz L. C. VandeVord P. J. Costa B. M. (2023). CNS4 causes subtype-specific changes in agonist efficacy and reversal potential of permeant cations in NMDA receptors. Pharmacol. Res. Perspect. 11, –e01107. doi: 10.1002/PRP2.1107, PMID: 37283007 PMC10245146

[ref7] Bond M. Rogers G. Peters J. Anderson R. Hoyle M. Miners A.. (2012). The effectiveness and cost-effectiveness of donepezil, galantamine, rivastigmine and memantine for the treatment of Alzheimer’s disease (review of Technology Appraisal No. 111): a systematic review and economic model. Health Technol. Assess. 16, 1–470. doi: 10.3310/hta16210, PMID: 22541366 PMC4780923

[ref8] Breijyeh Z. Karaman R. (2020). Comprehensive review on Alzheimer’s disease: causes and treatment. Molecules 25:5789. doi: 10.3390/molecules25245789, PMID: 33302541 PMC7764106

[ref9] Busche M. A. Hyman B. T. (2020). Synergy between amyloid-β and tau in Alzheimer’s disease. Nat. Neurosci. 23, 1183–1193. doi: 10.1038/s41593-020-0687-6 32778792 PMC11831977

[ref10] Caballero E. Hernando-Pérez E. Tapias V. Calvo-Rodríguez M. Villalobos C. Núñez L. (2022). Amyloid beta oligomers-induced Ca2+ entry pathways: role of neuronal networks, NMDA receptors and amyloid channel formation. Biomedicine 10:1153. doi: 10.3390/BIOMEDICINES10051153/S1, PMID: 35625890 PMC9138537

[ref11] Calvo M. Sanz-Blasco S. Caballero E. Villalobos C. Núñez L. (2015). Susceptibility to excitotoxicity in aged hippocampal cultures and neuroprotection by non-steroidal anti-inflammatory drugs: role of mitochondrial calcium. J. Neurochem. 132, 403–417. doi: 10.1111/JNC.13004, PMID: 25492611

[ref12] Calvo-Rodríguez M. de la Fuente C. García-Durillo M. García-Rodríguez C. Villalobos C. Núñez L. (2017). Aging and amyloid β oligomers enhance TLR4 expression, LPS-induced Ca2+ responses, and neuron cell death in cultured rat hippocampal neurons. J. Neuroinflammation 14:24. doi: 10.1186/S12974-017-0802-0, PMID: 28143556 PMC5282876

[ref13] Calvo-Rodriguez M. Garcia-Durillo M. Villalobos C. Nunez L. (2016). Aging enables Ca2+ overload and apoptosis induced by amyloid-β oligomers in rat hippocampal neurons: neuroprotection by non-steroidal anti-inflammatory drugs and R-flurbiprofen in aging neurons. J. Alzheimer’s Dis. 54, 207–221. doi: 10.3233/JAD-151189, PMID: 27447424

[ref14] Calvo-Rodriguez M. Hernando-Perez E. Nuñez L. Villalobos C. (2019). Amyloid β oligomers increase ER-mitochondria Ca2+ cross talk in young hippocampal neurons and exacerbate aging-induced intracellular Ca2+ remodeling. Front. Cell. Neurosci. 13:22. doi: 10.3389/fncel.2019.00022, PMID: 30800057 PMC6376150

[ref15] Carvajal F. J. Mattison H. A. Cerpa W. (2016). Role of NMDA receptor-mediated glutamatergic signaling in chronic and acute neuropathologies. Neural Plast. 2016:2701526. doi: 10.1155/2016/2701526, PMID: 27630777 PMC5007376

[ref16] Chayrov R. Volkova T. Perlovich G. Zeng L. Li Z. Štícha M.. (2022). Synthesis, neuroprotective effect and physicochemical studies of novel peptide and nootropic analogues of alzheimer disease drug. Pharmaceuticals (Basel, Switzerland) 15:1108. doi: 10.3390/PH15091108 36145329 PMC9500833

[ref17] Citron M. (2010). Alzheimer’s disease: Strategies for disease modification. Nat. Rev. Drug Discov. 9, 387–398. doi: 10.1038/nrd2896 20431570

[ref18] Coelho B. P. Gaelzer M. M. dos Santos Petry F. Hoppe J. B. Trindade V. M. T. Salbego C. G.. (2019). Dual effect of doxazosin: anticancer activity on SH-SY5Y neuroblastoma cells and neuroprotection on an *in vitro* model of Alzheimer’s disease. Neuroscience 404, 314–325. doi: 10.1016/j.neuroscience.2019.02.005 30771511

[ref19] Companys-Alemany J. Turcu A. L. Schneider M. Müller C. E. Vázquez S. Griñán-Ferré C.. (2022). NMDA receptor antagonists reduce amyloid-β deposition by modulating calpain-1 signaling and autophagy, rescuing cognitive impairment in 5XFAD mice. Cell. Mol. Life Sci. 79:408. doi: 10.1007/S00018-022-04438-4, PMID: 35810220 PMC9271115

[ref20] Correa B. H. M. Moreira C. R. Hildebrand M. E. Vieira L. B. (2022). The role of voltage-gated calcium channels in basal ganglia neurodegenerative disorders. Curr. Neuropharmacol. 21, 183–201. doi: 10.2174/1570159X20666220327211156, PMID: 35339179 PMC10190140

[ref21] Danysz W. Parsons C. G. (2012). Alzheimer’s disease, β-amyloid, glutamate, NMDA receptors and memantine – searching for the connections. Br. J. Pharmacol. 167, 324–352. doi: 10.1111/J.1476-5381.2012.02057.X, PMID: 22646481 PMC3481041

[ref22] Danysz W. Parsons C. G. Mobius H.-J. Stöffler A. Quack G. (2000). Neuroprotective and symptomatological action of memantine relevant for alzheimer’s disease — a unified glutamatergic hypothesis on the mechanism of action. Neurotox. Res. 2, 85–97. doi: 10.1007/bf03033787, PMID: 16787834

[ref23] de Wet S. Mangali A. Batt R. Kriel J. Vahrmeijer N. Niehaus D.. (2023). The highs and lows of memantine—an autophagy and mitophagy inducing agent that protects mitochondria. Cells 12:1726. doi: 10.3390/CELLS12131726/S1, PMID: 37443760 PMC10340721

[ref24] Delussi M. Sciruicchio V. Paolo T. Morgante F. Salvatore E. Ferrara I. P.. (2022). Lower prevalence of chronic pain in manifest Huntington’s disease: a pilot observational study. Brain Sci. 12:676. doi: 10.3390/BRAINSCI12050676, PMID: 35625062 PMC9139182

[ref25] Findley C. A. Bartke A. Hascup K. N. Hascup E. R. (2019). Amyloid beta-related alterations to glutamate signaling dynamics during Alzheimer’s disease progression. ASN Neuro 11:175909141985554. doi: 10.1177/1759091419855541, PMID: 31213067 PMC6582288

[ref26] Finke C. Kopp U. A. Prüss H. Dalmau J. Wandinger K. P. Ploner C. J. (2012). Cognitive deficits following anti-NMDA receptor encephalitis. J. Neurol. Neurosurg. Psychiatry 83, 195–198. doi: 10.1136/JNNP-2011-300411, PMID: 21933952 PMC3718487

[ref27] Folch J. Busquets O. Ettcheto M. Sánchez-López E. Castro-Torres R. D. Verdaguer E.. (2018). Memantine for the treatment of dementia: A review on its current and future applications. J. Alzheimers Dis. 62, 1223–1240. doi: 10.3233/JAD-170672, PMID: 29254093 PMC5870028

[ref28] Ghasemi M. Schachter S. C. (2011). The NMDA receptor complex as a therapeutic target in epilepsy: a review. Epilepsy Behav. 22, 617–640. doi: 10.1016/J.YEBEH.2011.07.024, PMID: 22056342

[ref29] Gonzalez J. Jurado-Coronel J. C. Ávila M. F. Sabogal A. Capani F. Barreto G. E. (2015). NMDARs in neurological diseases: A potential therapeutic target. Int. J. Neurosci. 125, 315–327. doi: 10.3109/00207454.2014.940941, PMID: 25051426

[ref30] Gorbachenko V. A. Lukyanetz E. A. (2020). Effects of memantine on the passive avoidance test in young rats. Fiziologichnyi Zhurnal 66, 3–10. doi: 10.15407/FZ66.05.003

[ref31] Görgülü A. Kinş T. Çobanoǧlu S. Ünal F. Izgi N. Yanik B.. (2000). Reduction of edema and infarction by Memantine and MK-801 after focal cerebral ischaemia and reperfusion in rat. Acta Neurochir. 142, 1287–1292. doi: 10.1007/s007010070027, PMID: 11201645

[ref32] Hanzha V. V. Rozumna N. M. Kravenska Y. V. Spivak M. Y. Lukyanetz E. A. (2023). The effect of cerium dioxide nanoparticles on the viability of hippocampal neurons in Alzheimer’s disease modeling. Front. Cell. Neurosci. 17:1131168. doi: 10.3389/FNCEL.2023.1131168/BIBTEX, PMID: 37006473 PMC10060808

[ref33] Harrill J. A. Chen H. Streifel K. M. Yang D. Mundy W. R. Lein P. J. (2015). Ontogeny of biochemical, morphological and functional parameters of synaptogenesis in primary cultures of rat hippocampal and cortical neurons. Mol. Brain 8, 1–15. doi: 10.1186/S13041-015-0099-9 25757474 PMC4339650

[ref34] Hassan N. A. Alshamari A. K. Hassan A. A. Elharrif M. G. Alhajri A. M. Sattam M.. (2022). Advances on therapeutic strategies for Alzheimer’s disease: from medicinal plant to nanotechnology. Molecules (Basel, Switzerland) 27:4839. doi: 10.3390/MOLECULES27154839 35956796 PMC9369981

[ref35] Hirano K. Fujimaki M. Sasazawa Y. Yamaguchi A. Ishikawa K. I. Miyamoto K.. (2019). Neuroprotective effects of memantine via enhancement of autophagy. Biochem. Biophys. Res. Commun. 518, 161–170. doi: 10.1016/J.BBRC.2019.08.025, PMID: 31431260

[ref36] Hooshmandi E. Moosavi M. Katinger H. Sardab S. Ghasemi R. Maghsoudi N. (2020). CEPO (carbamylated erythropoietin)-Fc protects hippocampal cells in culture against beta amyloid-induced apoptosis: considering Akt/GSK-3β and ERK signaling pathways. Mol. Biol. Rep. 47, 2097–2108. doi: 10.1007/s11033-020-05309-6, PMID: 32067159

[ref37] Ippoliti I. Ancidoni A. Da Cas R. Pierantozzi A. Vanacore N. Trotta F. (2023). Anti-dementia drugs: a descriptive study of the prescription pattern in Italy. Neurol. Sci. 44, 1587–1595. doi: 10.1007/S10072-022-06586-8, PMID: 36595207 PMC9807981

[ref38] Karapetyan G. Fereshetyan K. Harutyunyan H. Yenkoyan K. (2022). The synergy of β amyloid 1-42 and oxidative stress in the development of Alzheimer’s disease-like neurodegeneration of hippocampal cells. Sci. Rep. 12, –17883. doi: 10.1038/S41598-022-22761-5, PMID: 36284177 PMC9596457

[ref39] Kim C. Yousefian-Jazi A. Choi S. H. Chang I. Lee J. Ryu H. (2021). Non-cell autonomous and epigenetic mechanisms of Huntington’s disease. Int. J. Mol. Sci. 22, –12499. doi: 10.3390/IJMS222212499, PMID: 34830381 PMC8617801

[ref40] Kodis E. J. Choi S. Swanson E. Ferreira G. Bloom G. S. (2018). N-methyl-D-aspartate receptor–mediated calcium influx connects amyloid-β oligomers to ectopic neuronal cell cycle reentry in Alzheimer’s disease. Alzheimers Dement. 14, 1302–1312. doi: 10.1016/J.JALZ.2018.05.017, PMID: 30293574 PMC8363206

[ref41] Kolcheva M. Kortus S. Krausova B. H. Barackova P. Misiachna A. Danacikova S.. (2021). Specific pathogenic mutations in the M3 domain of the GluN1 subunit regulate the surface delivery and pharmacological sensitivity of NMDA receptors. Neuropharmacology 189:108528. doi: 10.1016/J.NEUROPHARM.2021.108528, PMID: 33773999

[ref42] Korol T. Y. Korol S. V. Kostyuk E. P. Kostyuk P. G. (2008). β-amyloid-induced changes in calcium homeostasis in cultured hippocampal neurons of the rat. Neurophysiology 40, 6–9. doi: 10.1007/s11062-008-9013-8

[ref43] Kravenska E. V. Chopovska V. V. Yavorskaya E. N. Lukyanetz E. A. (2012). The role of mitochondria in the development of Alzheimer’s disease. Tavrichesky Med. Biol. Bull. 15, 147–149.

[ref44] Kravenska E. V. Ganzha V. V. Yavorskaya E. N. Lukyanetz E. A. (2016). Effect of cyclosporin A on the viability of hippocampal cells cultured under conditions of modeling of Alzheimer’s disease. Neurophysiology 48, 246–251. doi: 10.1007/s11062-016-9595-5

[ref45] Kravenska Y. Nieznanska H. Nieznanski K. Lukyanetz E. Szewczyk A. Koprowski P. (2020). The monomers, oligomers, and fibrils of amyloid-β inhibit the activity of mitoBKCa channels by a membrane-mediated mechanism. Biochim. Biophys. Acta 1862:183337. doi: 10.1016/J.BBAMEM.2020.183337, PMID: 32380169

[ref46] Kruchenko Z. A. Gorbachenko V. A. Chereda I. S. Lukyanetz E. A. (2014). Effect of memantine on motor behavioral phenomena in rats of different ages. Neurophysiology 46, 448–451. doi: 10.1007/S11062-015-9472-7/METRICS

[ref47] Lacor P. N. Buniel M. C. Furlow P. W. Clemente A. S. Velasco P. T. Wood M.. (2007). Aβ oligomer-induced aberrations in synapse composition, shape, and density provide a molecular basis for loss of connectivity in Alzheimer’s disease. J. Neurosci. 27, 796–807. doi: 10.1523/JNEUROSCI.3501-06.2007, PMID: 17251419 PMC6672917

[ref48] Liu N. Li Y. Hong Y. Huo J. Chang T. Wang H.. (2023). Altered brain activities in mesocorticolimbic pathway in primary dysmenorrhea patients of long-term menstrual pain. Front. Neurosci. 17:1098573. doi: 10.3389/FNINS.2023.1098573/BIBTEX, PMID: 36793538 PMC9922713

[ref49] Lopes J. P. Tarozzo G. Reggiani A. Piomelli D. Cavalli A. (2013). Galantamine potentiates the neuroprotective effect of memantine against NMDA-induced excitotoxicity. Brain Behav. 3, 67–74. doi: 10.1002/brb3.118, PMID: 23532860 PMC3607148

[ref50] Majidazar R. Rezazadeh-Gavgani E. Sadigh-Eteghad S. Naseri A. (2022). Pharmacotherapy of Alzheimer’s disease: an overview of systematic reviews. Eur. J. Clin. Pharmacol. 78, 1567–1587. doi: 10.1007/S00228-022-03363-6, PMID: 35881170

[ref51] Majláth Z. Török N. Toldi J. Vécsei L. (2016). Memantine and kynurenic acid: current neuropharmacological aspects. Curr. Neuropharmacol. 14, 200–209. doi: 10.2174/1570159X14666151113123221, PMID: 26564141 PMC4825950

[ref52] Marotta G. Basagni F. Rosini M. Minarini A. (2020). Memantine derivatives as multitarget agents in Alzheimer’s disease. Molecules 25:4005. doi: 10.3390/molecules25174005, PMID: 32887400 PMC7504780

[ref53] Miguel-Hidalgo J. J. Alvarez X. A. Cacabelos R. Quack G. (2002). Neuroprotection by memantine against neurodegeneration induced by β-amyloid(1-40). Brain Res. 958, 210–221. doi: 10.1016/S0006-8993(02)03731-9, PMID: 12468047

[ref54] Mody I. MacDonald J. F. (1995). NMDA receptor-dependent excitotoxicity: the role of intracellular Ca2+ release. Trends Pharmacol. Sci. 16, 356–359. doi: 10.1016/S0165-6147(00)89070-7, PMID: 7491714

[ref55] Nakazawa K. Sapkota K. (2020). The origin of NMDA receptor hypofunction in schizophrenia. Pharmacol. Ther. 205:107426. doi: 10.1016/J.PHARMTHERA.2019.107426, PMID: 31629007 PMC6981256

[ref56] Napoli A. Obeid I. (2016). Comparative analysis of human and rodent brain primary neuronal culture spontaneous activity using micro-electrode array technology. J. Cell. Biochem. 117, 559–565. doi: 10.1002/JCB.25312, PMID: 26284690

[ref57] Olivares D. Deshpande V. K. Shi Y. Lahiri D. K. Greig N. H. Rogers J. T.. (2012). N-Methyl D-Aspartate (NMDA) receptor antagonists and memantine treatment for Alzheimer’s disease, vascular dementia and Parkinson’s disease. Curr. Alzheimer Res. 9, 746–758. doi: 10.2174/156720512801322564, PMID: 21875407 PMC5002349

[ref58] Padovani A. Falato S. Pegoraro V. (2023). Extemporaneous combination of donepezil and memantine to treat dementia in Alzheimer disease: evidence from Italian real-world data. Curr. Med. Res. Opin. 39, 567–577. doi: 10.1080/03007995.2023.2182530, PMID: 36803101

[ref59] Pendeliuk V. S. Melnick I. V. (2023). Excitatory synchronization of rat hippocampal interneurons during network activation *in vitro*. Front. Cell. Neurosci. 17:1129991. doi: 10.3389/FNCEL.2023.1129991, PMID: 36970420 PMC10034414

[ref60] Penke B. Szucs M. Bogár F. (2020). Oligomerization and conformational change turn monomeric β-amyloid and tau proteins toxic: Their role in Alzheimer’s pathogenesis. Molecules 25:1659. doi: 10.3390/molecules25071659, PMID: 32260279 PMC7180792

[ref61] Polyák H. Galla Z. Nánási N. Cseh E. K. Rajda C. Veres G.. (2023). The tryptophan-kynurenine metabolic system is suppressed in cuprizone-induced model of demyelination simulating progressive multiple sclerosis. Biomedicine 11:945. doi: 10.3390/BIOMEDICINES11030945/S1, PMID: 36979924 PMC10046567

[ref62] Rozumna N. M. Shkryl V. M. Ganzha V. V. Lukyanetz E. A. (2020). Effects of modeling of hypercalcemia and β-amyloid on cultured hippocampal neurons of rats. Neurophysiology 52, 348–357. doi: 10.1007/S11062-021-09891-8/METRICS

[ref63] Saeedi M. Mehranfar F. (2022). Challenges and approaches of drugs such as memantine, donepezil, rivastigmine, and aducanumab in the treatment, control and management of Alzheimer’s disease. Recent Pat. Biotechnol. 16, 102–121. doi: 10.2174/1872208316666220302115901, PMID: 35236274

[ref64] Sahu M. P. Nikkilä O. Lagas S. Kolehmainen S. Castrén E. (2019). Culturing primary neurons from rat hippocampus and cortex. Neuron. Signal. 3, –20180207. doi: 10.1042/NS20180207, PMID: 32714598 PMC7363313

[ref65] Shkryl V. M. (2022). The spatio-temporal properties of calcium transients in hippocampal pyramidal neurons *in vitro*. Front. Cell. Neurosci. 16:1054950. doi: 10.3389/FNCEL.2022.1054950/BIBTEX, PMID: 36589284 PMC9795003

[ref66] Tan C. C. Yu J. T. Wang H. F. Tan M. S. Meng X. F. Wang C.. (2014). Efficacy and safety of donepezil, galantamine, rivastigmine, and memantine for the treatment of Alzheimer’s disease: a systematic review and meta-analysis. J. Alzheimers Dis. 41, 615–631. doi: 10.3233/jad-132690, PMID: 24662102

[ref67] Tanaka M. Bohár Z. Martos D. Telegdy G. Vécsei L. (2020). Antidepressant-like effects of kynurenic acid in a modified forced swim test. Pharmacol. Rep. 72, 449–455. doi: 10.1007/S43440-020-00067-5/TABLES/1 32162182

[ref68] Tanaka M. Diano M. Battaglia S. (2023). Editorial: Insights into structural and functional organization of the brain: evidence from neuroimaging and non-invasive brain stimulation techniques. Front. Psych. 14:1225755. doi: 10.3389/FPSYT.2023.1225755/BIBTEX, PMID: 37377471 PMC10291688

[ref69] Tanaka M. Spekker E. Szabó Á. Polyák H. Vécsei L. (2022a). Modelling the neurodevelopmental pathogenesis in neuropsychiatric disorders. Bioactive kynurenines and their analogues as neuroprotective agents—in celebration of 80th birthday of Professor Peter Riederer. J. Neural Transm. 129, 627–642. doi: 10.1007/S00702-022-02513-5, PMID: 35624406

[ref70] Tanaka M. Szabó Á. Vécsei L. (2022b). Integrating armchair, bench, and bedside research for behavioral neurology and neuropsychiatry: editorial. Biomedicine 10:2999. doi: 10.3390/BIOMEDICINES10122999, PMID: 36551755 PMC9775182

[ref71] Thakral S. Yadav A. Singh V. Kumar M. Kumar P. Narang R.. (2023). Alzheimer’s disease: Molecular aspects and treatment opportunities using herbal drugs. Ageing Res. Rev. 88:101960. doi: 10.1016/J.ARR.2023.101960, PMID: 37224884

[ref72] Tyshchenko Y. M. Lukyanetz E. A. (2017). Effects of memantine on behavioral indices of rats in the open field. Neurophysiology 49, 453–457. doi: 10.1007/S11062-018-9708-4/METRICS

[ref73] Tyshchenko Y. N. Lukyanetz E. A. (2020). The role of beta-amyloid in norm and at Alzheimer’s disease. Fiziol. Zh. 66, 88–96. doi: 10.15407/FZ66.06.088

[ref74] Vicario-Abejón C. (2004). Long-term culture of hippocampal neurons. Curr. Protoc. Neurosci. 26, 3.2.1–3.2.13. doi: 10.1002/0471142301.NS0302S26 18428599

[ref75] Volbracht C. Van Beek J. Zhu C. Blomgren K. Leist M. (2006). Neuroprotective properties of memantine in different *in vitro* and *in vivo* models of excitotoxicity. Eur. J. Neurosci. 23, 2611–2622. doi: 10.1111/j.1460-9568.2006.04787.x, PMID: 16817864

[ref76] Wang R. Reddy P. H. (2017). Role of Glutamate and NMDA Receptors in Alzheimer’s Disease. J. Alzheimers Dis. 57, 1041–1048. doi: 10.3233/JAD-160763, PMID: 27662322 PMC5791143

[ref77] Wilcox M. R. Nigam A. Glasgow N. G. Narangoda C. Phillips M. B. Patel D. S.. (2022). Inhibition of NMDA receptors through a membrane-to-channel path. Nat. Commun. 13:4114. doi: 10.1038/S41467-022-31817-Z, PMID: 35840593 PMC9287434

[ref78] Wu Q. J. Tymianski M. (2018). Targeting NMDA receptors in stroke: new hope in neuroprotection. Mol. Brain 11, 15–14. doi: 10.1186/S13041-018-0357-8, PMID: 29534733 PMC5851248

[ref79] Xu L. Z. Li B. Q. Li F. Y. Li Y. Qin W. Zhao Y.. (2023). NMDA receptor GluN2B Subunit is involved in excitotoxicity mediated by death-associated protein kinase 1 in Alzheimer’s disease. J. Alzheimer’s Dis. 91, 877–893. doi: 10.3233/JAD-220747, PMID: 36502323

[ref80] Yang Q. Ke Y. Luo J. Tang Y. (2017). Protocol for culturing low density pure rat hippocampal neurons supported by mature mixed neuron cultures. J. Neurosci. Methods 277, 38–45. doi: 10.1016/J.JNEUMETH.2016.12.002, PMID: 27956052

[ref81] Yavorsky V. A. Rozumna N. M. Lukyanetz E. A. (2023). Influence of amyloid beta on impulse spiking of isolated hippocampal neurons. Front. Cell. Neurosci. 17:1132092. doi: 10.3389/FNCEL.2023.1132092/BIBTEX, PMID: 37124394 PMC10133472

[ref82] Yu S. P. Jiang M. Q. Shim S. S. Pourkhodadad S. Wei L. (2023). Extrasynaptic NMDA receptors in acute and chronic excitotoxicity: implications for preventive treatments of ischemic stroke and late-onset Alzheimer’s disease. Mol. Neurodegener. 18, 43–29. doi: 10.1186/S13024-023-00636-1, PMID: 37400870 PMC10318843

[ref83] Zhang H. Li X. Wang X. Xu J. Elefant F. Wang J. (2023). Cellular response to β-amyloid neurotoxicity in Alzheimer’s disease and implications in new therapeutics. Anim. Models Exp. Med. 6, 3–9. doi: 10.1002/ame2.12313, PMID: 36872303 PMC9986234

