# Supplementary figures and images for: Memantine protects the cultured rat hippocampal neurons treated by NMDA and amyloid β1–42
Source: Front Neurosci. 2023 Dec 8;17:1269664. doi: 10.3389/fnins.2023.1269664 (PMC10748420; doi:10.3389/fnins.2023.1269664)

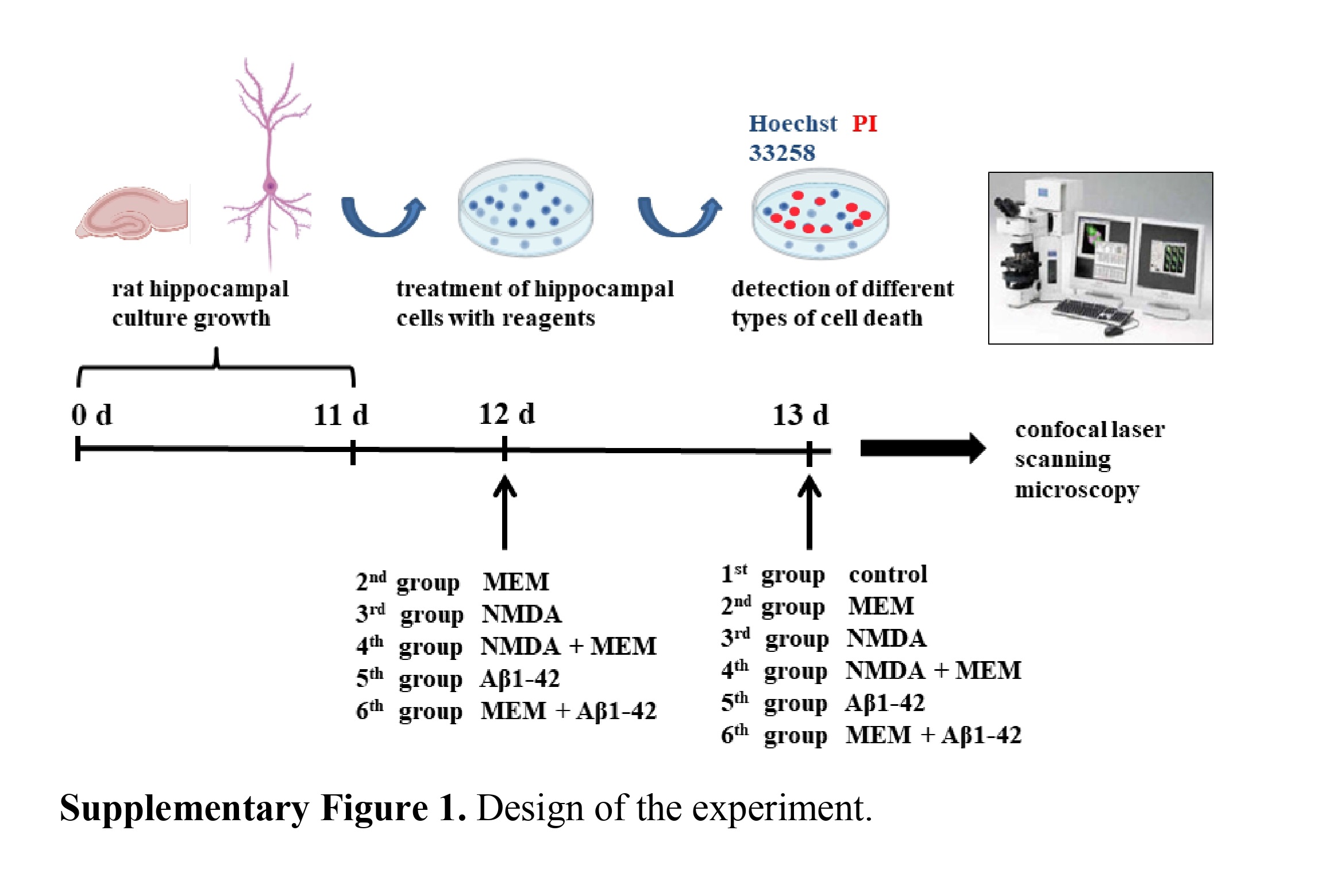

Supplement: Supplementary file 1 [file Image_1.jpg]
